# Supplementary material for: Relative Binding Free Energy Estimation of Congeneric Ligands and Macromolecular Mutants with the Alchemical Transfer Method with Coordinate Swapping
Source: J Chem Inf Model. 2025 Mar 26;65(7):3706–14. doi: 10.1021/acs.jcim.5c00207 (PMC12004517; doi:10.1021/acs.jcim.5c00207)
Supplement: Supplementary file 1 — ci5c00207_si_001.pdf [file ci5c00207_si_001.pdf]

Supplementary Information for: Relative Binding Free Energy Estimation of Congeneric Ligands and Macromolecular Mutants with the Alchemical Transfer Method with Coordinate Swapping

Emilio Gallicchio[*,†,‡,¶]

†*Department of Chemistry and Biochemistry, Brooklyn College of the City University of New York, New York, NY, 11210*

‡*Ph.D. Program in Chemistry, The Graduate Center of the City University of New York, New York, NY, 10016*

¶*Ph.D. Program in Biochemistry, The Graduate Center of the City University of New York, New York, NY, 10016*

E-mail: egallicchio@brooklyn.cuny.edu

This supplementary document contains the numerical results for the TYK2 protein-ligand benchmark set and the proof of the coordinate swapping protocol.

TYK-2 Protein-Ligand Relative Binding Free Energies

Table S.1: The relative binding free energy estimates of the TYK2 pairs using the standard alchemical transfer (ATM-RBFE) and coordinate swapping (ATS-RBFE) workflows compared to the corresponding differences of experimental affinities.

Ligand Pair	$\Delta\Delta G_b{}^{a,b}$ (ATM-RBFE)	$\Delta\Delta G_b{}^{a,b}$ (ATS-RBFE)	$\Delta\Delta G_b{}^{a,c}$ (Expt)
jmc23-ejm55	0.45 ± 0.26	0.44 ± 0.21	2.49
ejm44-ejm55	-2.55 ± 0.27	-2.25 ± 0.23	-1.79
ejm49-ejm31	-1.47 ± 0.28	-1.43 ± 0.24	-1.79
ejm31-ejm46	-0.82 ± 0.26	-0.80 ± 0.20	-1.77
jmc28-jmc27	-0.25 ± 0.26	-0.40 ± 0.20	-0.30
ejm42-ejm48	0.82 ± 0.27	0.01 ± 0.22	0.78
ejm31-ejm43	0.68 ± 0.27	1.21 ± 0.21	1.28
ejm50-ejm42	-0.91 ± 0.26	-0.78 ± 0.20	-0.80
ejm42-ejm55	-0.86 ± 0.26	-0.19 ± 0.19	0.57
jmc23-ejm46	-0.38 ± 0.26	-0.40 ± 0.20	0.39
ejm31-ejm45	0.28 ± 0.27	0.33 ± 0.23	-0.02
ejm55-ejm54	-0.32 ± 0.27	-0.44 ± 0.23	-1.32
ejm45-ejm42	0.21 ± 0.27	0.27 ± 0.21	-0.22
ejm31-jmc28	0.19 ± 0.27	-0.27 ± 0.22	-1.44
ejm31-ejm48	0.79 ± 0.28	0.31 ± 0.24	0.54
ejm47-ejm31	-0.76 ± 0.27	-0.19 ± 0.22	0.16
ejm47-ejm55	-0.94 ± 0.27	-0.73 ± 0.21	0.49
ejm44-ejm42	-2.64 ± 0.27	-2.21 ± 0.22	-2.36
jmc23-jmc27	-0.79 ± 0.26	-0.43 ± 0.20	0.42
ejm43-ejm55	-1.75 ± 0.26	-1.48 ± 0.21	-0.95
jmc23-jmc30	-0.60 ± 0.27	-1.10 ± 0.21	0.76
jmc28-jmc30	-0.74 ± 0.27	-1.29 ± 0.22	0.04
ejm42-ejm54	-0.13 ± 0.27	-0.20 ± 0.22	-0.75
ejm49-ejm50	-1.08 ± 0.28	-0.84 ± 0.25	-1.23
RMSD^d vs. ATM-RBFE		0.37	
R^e vs. ATM-RBFE		0.91	
RMSD^d vs. Expt	0.93	0.87	
R^e vs. Expt	0.65	0.68	

^{a}In kcal/mol. ^{b}One standard deviation uncertainties in parenthesis. ^{c}From reference 1. ^{d} Root mean square deviation in kcal/mol. ^{e} Correlation coefficient.

Proof of the coordinate swapping relative binding free energy formula

The statistical mechanics expression for the bimolecular binding constant between a receptor R and a ligand A is[2–4]

$$K_b(A) = \frac{C^\circ}{8\pi^2}\frac{z_{RA}}{z_R z_A}, \tag{12}$$

where z_{RA}, z_R, and z_A, the internal partition functions of the complex, receptor, and ligand, respectively, are defined as

$$z_A = \int dx_A e^{-\beta\Psi_A(x_A)} \tag{13}$$

and similarly for R,

$$z_{RA} = \int dx_R dx_A d\zeta_A I(\zeta_A) e^{-\beta\Psi_{RA}(x_R, x_A, \zeta_A)} \tag{14}$$

where x_R and x_A are the internal coordinates of the receptor and ligand respectively. $\zeta_A = (\mathbf{c}_A, \omega_A)$ denotes, collectively, the three position and three orientation coordinates of ligand A relative to the reference frame of the receptor, Ψ_A and Ψ_{RA} denote the effective potential energy functions in the solvent potential of mean force representation[4,5] of the ligand in the solvent and of the ligand bound to the solvated receptor, respectively. The function $I(\zeta_A)$ is defined later. The function Ψ is the potential energy function of the system in the solvent potential of mean force representation, obtained by pre-averaging over the solvent degrees of freedom. The potential of mean force representation does not introduce approximations; it is used here and in the following derivations as for notational convenience to avoid explicitly listing the coordinates of the solvent.

In Eq. (14), the function $I(\zeta_A)$ in Eq. (14) is an indicator function that is set to 1 if the position and orientation of the ligand are such that receptor and ligand are considered bound, and zero otherwise.[2,3,6] The volume of configurational space encompassed by the

indicator function is denoted here by $V_{\rm site}\Omega_{\rm site}$:

$$\int d\zeta I(\zeta) = V_{\rm site}\Omega_{\rm site} \tag{15}$$

For later use, we consider also an indicator function $I^*(\zeta)$ identical to the one that defines the complex, but centered at a point $\mathbf{d}$ in the solvent bulk relative to the reference frame of the receptor.

Consider now the ratio, $K_b(\mathrm{B})/K_b(\mathrm{A})$, of the equilibrium binding constants of two ligands A and B to the same receptor R in the same binding mode described by the indicator function $I(\zeta)$. To simplify the notation, in the following we will also assume that $I(\zeta)$ is independent of the orientation coordinates. That is we assume that $I(\zeta) = I(\mathbf{c})$, where $\mathbf{c}$ is a chosen centroid of the ligand. Under this assumption $\Omega_{\rm site} = 8\pi^2$ which cancels the same factor in the denominator of Eq. (12). The extension of this derivation to orientation-dependent binding mode definitions is straightforward and does not affect the end result.

From Eq. (12) and canceling the common factor $z_{\rm R}$, we have

$$\frac{K_b(\mathrm{B})}{K_b(\mathrm{A})} = \frac{z_{\rm RB} z_{\rm A}}{z_{\rm RA} z_{\rm B}}\,, \tag{16}$$

where $z_{\rm RB}$ is the intramolecular configurational partition function of the complex between R and B, $z_{\rm B}$ is the intramolecular partition function of ligand B, and similarly for $z_{\rm RA}$ and $z_{\rm A}$. To express Eq. (16) as an ensemble average the products $z_{\rm RB} z_{\rm A}$ and $z_{\rm RA} z_{\rm B}$ are expressed as partition function integrals of systems containing the receptor and the two ligands such that one of the ligands is in the binding site and the other is centered on a position of the bulk at distance $\mathbf{d}$ relative to the receptor coordinate frame. Specifically, we multiply the numerator of Eq. (16) by Eq. (15) for A and the denominator by the corresponding integral for B. The result is

$$\frac{K_b(B)}{K_b(A)} = \frac{\int dx_R dx_A dx_B d\mathbf{c}_A d\omega_A d\mathbf{c}_B d\omega_B I^*(\mathbf{c}_A) I(\mathbf{c}_B) e^{-\beta\Psi(x_R,x_A,x_B,\mathbf{c}_A,\omega_A,\mathbf{c}_B,\omega_B)}}{\int dx_R dx_A dx_B d\mathbf{c}_A d\omega_A d\mathbf{c}_B d\omega_B I(\mathbf{c}_A) I^*(\mathbf{c}_B) e^{-\beta\Psi(x_R,x_A,x_B,\mathbf{c}_A,\omega_A,\mathbf{c}_B,\omega_B)}} \tag{17}$$

Next, we express the integration variables in terms of the Cartesian coordinates of the atoms $\mathbf{r}_A$ calculated from the internal coordinates x_A, the centroid coordinates $\mathbf{c}_A$, and the orientational coordinates ω_A, and similarly for B. The centroids becomes a function of the ligands' Cartesian coordinates, which are constrained to be within the region spanned by each indicator function. In the denominator for example, the center of mass of B is constrained by $I^*()$, which is located in the solvent bulk. Hence, the coordinates of B are constrained to be near this location.

$$\frac{K_b(B)}{K_b(A)} = \frac{\int dx_R d\mathbf{r}_A d\mathbf{r}_B I^*(\mathbf{c}_A) I(\mathbf{c}_B) e^{-\beta\Psi(x_R,\mathbf{r}_A,\mathbf{r}_B)}}{\int dx_R d\mathbf{r}_A d\mathbf{r}_B I(\mathbf{c}_A) I^*(\mathbf{c}_B) e^{-\beta\Psi(x_R,\mathbf{r}_A,\mathbf{r}_B)}} \tag{18}$$

Next, we divide the atoms of the ligands into common subsets A' and B' and variable subsets A'' and B''. That is, for example, $\mathbf{r}_A = (\mathbf{r}_{A'}, \mathbf{r}_{A''})$, where $\mathbf{r}_{A'}$ are the coordinates of the atoms in the common subset of A and $\mathbf{r}_{A''}$ those of the atoms in the variable subset, and similarly for ligand B. The common subsets for A and B have the same dimension. Additionally, a one-to-one and invertible (bijective) mapping relationship is established between the atoms in the common subset of A and the corresponding atoms of B. The atoms in the variable subsets are assumed to belong to two corresponding sidechain of the molecule each attached to an anchoring atom, a of A and b for B, belonging to the common subset and mapped into each other.

Finally, to express Eq. (17) as an ensemble average, we perform the following change of variables in the integral at the numerator of Eq. (18): (i) displacement for the atoms in the variable subsets by the vector distance, $\mathbf{d}_{BA} = \mathbf{r}_b - \mathbf{r}_a \simeq \mathbf{d}$ between the two anchoring atoms, and

$$\begin{aligned} \mathbf{r}_{A''} &\rightarrow \mathbf{r}_{A''} + \mathbf{d}_{BA} \\ \mathbf{r}_{B''} &\rightarrow \mathbf{r}_{B''} - \mathbf{d}_{BA} \end{aligned} \tag{19}$$

(ii) swapping of the coordinates of the corresponding atoms in the two common subsets

$$\begin{aligned} \mathbf{r}_{A'} &\rightarrow \mathbf{r}_{B'} \\ \mathbf{r}_{B'} &\rightarrow \mathbf{r}_{A'} \end{aligned} \tag{20}$$

which in overall move the coordinates of B from the solvent to the binding site and the coordinates of A from the binding site to the solvent, but maintaining the internal coordinates of the common regions unchanged.

As a result of the transformations above, the centroid of A, forced to be within the solvent region by the indicator function $I^*()$, is moved to the solvent region corresponding to the indicator function $I()$. Assuming that $I()$ is large enough so that a centroid in one region always lands in the allowed region of the other as a result of the transformations and viceversa, the value of the product $I^*(\mathbf{c}_A)I(\mathbf{c}_B)$ is not affected by the transformation. Furthermore, because after the transformation approximately $I^*(\mathbf{c}_A) \rightarrow I^*(\mathbf{c}_A + \mathbf{d}) = I(\mathbf{c}_A)$, and similarly for B, this term becomes $I(\mathbf{c}_A)I^*(\mathbf{c}_B)$, matching the same term in the integral at the denominator of Eq. (18).

The absolute value of the determinant $|J|$ of the Jacobian of the variable transformations (19) and (20) is 1. This can shown by considering that the gradient of the coordinates resulting from the swapping transformation (20) relative to the original coordinates is a vector of zeros except for the term corresponding to the mapped atom, where it is 1. Similarly, the gradient for the displacement transformation (19) is 1 on the diagonal and 1 or -1 in correspondence with the anchoring atom. Finally, the transformation does not affect the coordinates of the receptor atoms, which are represented by an identity matrix block of the Jacobian.

For example, consider a system composed of a receptor with two atoms, a ligand A with four atoms, two in the common subset and two in the variable subset, and a ligand B made of three atoms with two in the common subset and one in the variable subset. Without loss

of generality, we index the atoms of the system so that the receptor is listed first followed by the ligand atoms so that the two corresponding anchoring atoms have the last two indexes (8 and 9). Ligand A has atoms 3 and 8 in the common subset and atoms 4 and 5 in the variable subset and ligand B has atoms 6 and 9 in the common subset and atom 7 in the variable subset. Atom 3 is mapped to atom 6 and atom 8 is mapped to atom 9. This arrangement results in the following coordinate transformation

$$
\begin{aligned}
\mathbf{r}'_1 &= \mathbf{r}_1 \\
\mathbf{r}'_2 &= \mathbf{r}_2 \\
\mathbf{r}'_3 &= \mathbf{r}_6 \\
\mathbf{r}'_4 &= \mathbf{r}_4 + \mathbf{r}_9 - \mathbf{r}_8 \\
\mathbf{r}'_5 &= \mathbf{r}_5 + \mathbf{r}_9 - \mathbf{r}_8 \\
\mathbf{r}'_6 &= \mathbf{r}_3 \\
\mathbf{r}'_7 &= \mathbf{r}_7 - \mathbf{r}_9 + \mathbf{r}_8 \\
\mathbf{r}'_8 &= \mathbf{r}_9 \\
\mathbf{r}'_9 &= \mathbf{r}_8
\end{aligned}
$$

where the primed symbols are the coordinates after the transformation. The Jacobian matrix $\partial \mathbf{r}'_i / \partial \mathbf{r}_j$ of the transformation above is (we show only the components for one of the

coordinate axis since the coordinate transformation does not include mixed terms)

$$\begin{pmatrix} 1 & 0 & 0 & 0 & 0 & 0 & 0 & 0 & 0 \\ 0 & 1 & 0 & 0 & 0 & 0 & 0 & 0 & 0 \\ 0 & 0 & 0 & 0 & 0 & 1 & 0 & 0 & 0 \\ 0 & 0 & 0 & 1 & 0 & 0 & 0 & -1 & 1 \\ 0 & 0 & 0 & 0 & 1 & 0 & 0 & -1 & 1 \\ 0 & 0 & 1 & 0 & 0 & 0 & 0 & 0 & 0 \\ 0 & 0 & 0 & 0 & 0 & 0 & 1 & 1 & -1 \\ 0 & 0 & 0 & 0 & 0 & 0 & 0 & 0 & 1 \\ 0 & 0 & 0 & 0 & 0 & 0 & 0 & 1 & 0 \end{pmatrix}$$

Because the swapping transformation is one-to-one, it is always possible to swap the columns of the Jacobian matrix so that each column has a 1 in the diagonal corresponding to either the original atom (displacement) or the mapped atom (swapping). This involves swapping columns 8 and 9 that correspond to the anchoring atoms. However, because the non-diagonal terms of these columns are above the diagonal ones (because the anchoring atoms affect the transformations of only the atoms with lower index), these terms remain in the upper triangular portion of the Jacobian matrix. The result of swapping the columns, which does

not change the absolute value of the determinant, yields

$$\begin{pmatrix} 1 & 0 & 0 & 0 & 0 & 0 & 0 & 0 & 0 \\ 0 & 1 & 0 & 0 & 0 & 0 & 0 & 0 & 0 \\ 0 & 0 & 1 & 0 & 0 & 0 & 0 & 0 & 0 \\ 0 & 0 & 0 & 1 & 0 & 0 & 0 & 1 & -1 \\ 0 & 0 & 0 & 0 & 1 & 0 & 0 & 1 & -1 \\ 0 & 0 & 0 & 0 & 0 & 1 & 0 & 0 & 0 \\ 0 & 0 & 0 & 0 & 0 & 0 & 1 & -1 & 1 \\ 0 & 0 & 0 & 0 & 0 & 0 & 0 & 1 & 0 \\ 0 & 0 & 0 & 0 & 0 & 0 & 0 & 0 & 1 \end{pmatrix}$$

The matrix above is an upper triangular matrix in which the diagonal blocks are identity matrices with unitary determinant and therefore its determinant is 1.[7]

With the preparation above, Eq. (18) is rewritten as:

$$\frac{K_b(B)}{K_b(A)} = \frac{\int dx_R d\mathbf{r}_{A'} d\mathbf{r}_{A''} d\mathbf{r}_{B'} d\mathbf{r}_{B''} I(\mathbf{c}_A) I^*(\mathbf{c}_B) e^{-\beta\Psi(x_R, \mathbf{r}_{B'}, \mathbf{r}_{A''}+\mathbf{d_{BA}}, \mathbf{r}_{A'}, \mathbf{r}_{B''}-\mathbf{d}_{BA})}}{\int dx_R d\mathbf{r}_{A'} d\mathbf{r}_{A''} d\mathbf{r}_{B'} d\mathbf{r}_{B''} I(\mathbf{c}_A) I^*(\mathbf{c}_B) e^{-\beta\Psi(x_R, \mathbf{r}_{A'}, \mathbf{r}_{A''}, \mathbf{r}_{B'}, \mathbf{r}_{B''})}} \tag{21}$$

Finally, Eq. (8) in the main text is recovered by multiplying and dividing the integrand in the numerator by the Boltzmann factor in the denominator.

Gradients of the Alchemical Potential Energy Function

The gradient of the potential in Eq. (3) in the main text with respect to the coordinate $\mathbf{r}_k$ of an atom is

$$\frac{\partial U_\lambda}{\partial \mathbf{r}_k} = \frac{\partial U_{RA+B}}{\partial \mathbf{r}_k} + W'(u)\frac{\partial u}{\partial \mathbf{r}_k} \tag{22}$$

where $W'(u)$ is the derivative of the alchemical perturbation function (4) in the main text, and the unperturbed potential energy function U_{RA+B}, the perturbed potential energy function U_{RB+A}, and the perturbation energy u are defined by Eqs. (9)–(11) in the main text.

Eq. (22) is then expressed as a linear combination of the gradients of the perturbed and unperturbed potentials:

$$\frac{\partial U_\lambda}{\partial \mathbf{r}_k} = [1 - W'(u)]\frac{\partial U_{RA+B}}{\partial \mathbf{r}_k} + W'(u)\frac{\partial U_{RB+A}}{\partial \mathbf{r}_k} \tag{23}$$

The gradient of the unperturbed potential energy function is collected by the molecular dynamics engine as usual. The perturbed potential energy function is the unperturbed one with the transformed coordinates (displaced and swapped),

$$U_{RB+A}(\mathbf{r}) = U_{RA+B}[\mathbf{r}'(\mathbf{r})] \tag{24}$$

hence, its gradients can be found by the chain rule

$$\frac{\partial U_{RB+A}}{\partial \mathbf{r}_k} = \sum_j \frac{\partial U_{RA+B}(\mathbf{r}')}{\partial \mathbf{r}'_j}\frac{\partial \mathbf{r}'_j}{\partial \mathbf{r}_k} \tag{25}$$

The first term in the sum above is the gradient of the system's potential energy function computed after the variable transformation, also available from the molecular dynamics engine.

If k refers to one of the atoms in the common regions of A' or B' other than the anchoring atom, only the coordinates of its mapped atom, k', in the transformed system depend on it. Hence, only the $j = k'$ term in the sum (25) is not zero. Furthermore because $\mathbf{r}'_{k'} = \mathbf{r}_k$, $\partial \mathbf{r}'_{k'}/\partial \mathbf{r}_k$ is the identity matrix. So we have:

$$\frac{\partial U_{RB+A}}{\partial \mathbf{r}_k} = \frac{\partial U_{RA+B}(\mathbf{r}')}{\partial \mathbf{r}'_{k'}} \quad k \in A', B' \tag{26}$$

which states that the gradient of U_{RB+A} with respect to atom k is the gradient of the potential energy of the transformed system with respect to its mapped atom.

Let us now consider an atom k in the variable region A'' of ligand A. In the transformed

system, atom k is translated by the displacement vector of the two anchoring atoms, $\mathbf{r}'_k = \mathbf{r}_k + \mathbf{r}_b - \mathbf{r}_a$, and because neither the positions of the anchoring atoms nor the coordinates of any other atom depend on it:

$$\frac{\partial U_{RB+A}}{\partial \mathbf{r}_k} = \frac{\partial U_{RA+B}(\mathbf{r}')}{\partial \mathbf{r}'_k} \quad k \in A'', B'' \tag{27}$$

where we observed that the same argument applies to one of the atoms in the variable region of B.

Finally, consider the anchoring atom of A, i.e. $k = a$ in Eq. (25). All of the transformed coordinates of the atoms in the variable regions of A and B depend on it through the displacement vector, $\mathbf{d}_{BA} = \mathbf{r}_b - \mathbf{r}_a$ for atoms in A'' and the opposite displacement vector for atoms in B''. Moreover, $\mathbf{r}'_b = \mathbf{r}_a$ because a in the common region of A. It follows that the terms $j = b$, and those for $j \in A''$ (with a negative sign) and for $j \in B''$ (with a positive sign) are not zero in Eq. (25):

$$\frac{\partial U_{RB+A}}{\partial \mathbf{r}_a} = \frac{\partial U_{RA+B}(\mathbf{r}')}{\partial \mathbf{r}'_b} + \sum_{j \in B''} \frac{\partial U_{RA+B}(\mathbf{r}')}{\partial \mathbf{r}'_j} - \sum_{j \in A''} \frac{\partial U_{RA+B}(\mathbf{r}')}{\partial \mathbf{r}'_j} \tag{28}$$

The same applies to the anchoring atom b, but with the sign reversed:

$$\frac{\partial U_{RB+A}}{\partial \mathbf{r}_b} = \frac{\partial U_{RA+B}(\mathbf{r}')}{\partial \mathbf{r}'_a} - \sum_{j \in B''} \frac{\partial U_{RA+B}(\mathbf{r}')}{\partial \mathbf{r}'_j} + \sum_{j \in A''} \frac{\partial U_{RA+B}(\mathbf{r}')}{\partial \mathbf{r}'_j} \tag{29}$$

In our implementation, the sums of the gradients in Eqs. (28) and (29) above are collected when scanning the gradients of the atoms. If an atom j is part of one of the variable regions, its gradient is added to those of the anchoring atoms with the correct sign depending on whether the atom belongs to A'' or B'' and whether the gradient of anchoring atom a or b is being updated.

References

(1) Ross, G. A.; Lu, C.; Scarabelli, G.; Albanese, S. K.; Houang, E.; Abel, R.; Harder, E. D.; Wang, L. The maximal and current accuracy of rigorous protein-ligand binding free energy calculations. *Communications Chemistry* **2023**, *6*, 222.

(2) Gilson, M. K.; Given, J. A.; Bush, B. L.; McCammon, J. A. The Statistical-Thermodynamic Basis for Computation of Binding Affinities: A Critical Review. *Biophys. J.* **1997**, *72*, 1047–1069.

(3) Gallicchio, E.; Levy, R. M. Recent Theoretical and Computational Advances for Modeling Protein-Ligand Binding Affinities. *Adv. Prot. Chem. Struct. Biol.* **2011**, *85*, 27–80.

(4) Gallicchio, E. In *Computational Peptide Science: Methods and Protocols*; Simonson, T., Ed.; Methods in Molecular Biology; Springer Nature, 2022; pp 303–334.

(5) Roux, B.; Simonson, T. Implicit Solvent Models. *Biophys. Chem.* **1999**, *78*, 1–20.

(6) Boresch, S.; Tettinger, F.; Leitgeb, M.; Karplus, M. Absolute binding free energies: A quantitative approach for their calculation. *J. Phys. Chem. B* **2003**, *107*, 9535–9551.

(7) Taboga, M. *Lectures on Matrix Algebra*; Statlect: https://www.statlect.com/matrix-algebra/block-matrix, 2021; Chapter Determinant of a Block Matrix.
